# A New Self-Reported Assessment Measure for COVID-19 Anxiety Scale (CDAS) in Iran: A Web-Based Study

**DOI:** 10.18502/ijph.v49i7.3585

**Published:** 2020-07

**Authors:** Ahmad ALIPOUR, Abolfazl GHADAMI, Aida FARSHAM, Negin DORRI

**Affiliations:** 1. Department of Psychology, Payame Noor University, Tehran, Iran; 2. Department of Assessment and Measurement, Allameh Tabatabai University, Tehran, Iran; 3. Department of Psychology, School of Health Psychology, University of Tehran, Tehran, Iran

**Keywords:** Coronavirus, Anxiety, Self-report, Iran

## Abstract

**Background:**

Given the epidemic of Corona disease and its associated anxiety, it is necessary to develop a tool to measure anxiety. This study was conducted to instruct Corona Disease Anxiety Scale (CDAS) to measure the level of anxiety, during the prevalence of the COVID-19 in Iran.

**Methods:**

The present study was considered as applied research in terms of purpose and descriptive-correlational research in terms of methodological. 318 individuals (aged from 18 to 60 years old) completed the Corona Disease Anxiety Scale (CDAS) and the General Health Questionnaire (GHQ) online

**Results:**

Corona Disease Anxiety Scale had a good internal consistency (*α*=0.91) and good convergent validity, correlating with the GHQ-28 (*r*=0.49, *P*>0.01). Exploratory analysis revealed psychological and physical factors. These 2 factor account for 51% of the total variance and 9 items were loaded on every factor.

**Conclusion:**

This scale is reliable and valid scale for measuring Corona anxiety in non-clinical Iranian population.

## Introduction

Coronaviruses are a large group of viruses that may cause infections such as a common cold to more severe diseases like MERS and SARS. Recently this virus called COVID-19. The recent virus outbreak began in Wuhan, people republic of China, in Dec 2019 (1), and within a short time, it has spread too many countries including the Islamic Republic of Iran.

In 2012 Middle East Respiratory Syndrome (MERS) was first reported in Saudi Arabia and has since spread to several other countries in the Middle East and it is known as MERS and is viral respiratory illness. Another illness related coronavirus is called Severe Acute Respiratory Syndrome (SARS) which counts as virus-related respiratory illness called SARS-associated corona-virus (SARS-CoV). In Feb 2003 the first reported case was in Asia. According to data the spread of SARS was in more than two dozen countries naming North America, South America, Europe, and Asia. The virus that caused COVID-19 spread fast and easy and many affected geographic areas. The spread of the virus means that it has infected the whole range of individuals and communities (2).

The range of illness with COVID-19 is from mild to severe. The main signs and symptoms of infections are known to be fever, cough, and difficulty breathing in no particular order (2).

The lack of information on the virus, its unknown treatment, it is pandemic in many countries and its high mortality rate, COVID-19’s raises many mental health problems and unpleasant emotions such as anxiety. Fear of the unknown is a human instinct intended for protecting our kind from potential threats (3). To this day, people are looking for more ways to relieve their anxiety because feelings such as Anxiety can make one unable to identify the real issues and it might affect one’s right decision making when it’s most needed (4).

Another problem with emotions such as anxiety and stress is that it can weaken a person's immune system and make one more at risk than a person without such emotions to the disease (5). Anxiety can significantly decrease people’s quality of life. Anxiety had a negative effect on health-related quality of life and self-reported functional limitations (6). As a result, people need to learn strategies to cope with anxiety on daily bases. Given the rapid spread of the disease and its lack of research in this regard, researching to help identify the disease, and the anxiety it creates, are essential and can help improve the quality of life of individuals.

Anxiety measuring instruments have been developed for major health-threatening diseases such as SARS and MERS and other respiratory diseases. So far as information is available, there are no instruments created for coronavirus because it is new. In this study, we attempted to develop an instrument for measuring and screening anxiety in the pandemic of Corona.

## Methods

The present study was considered as applied research in terms of purpose and descriptive-correlational research in terms of methodological. The statistical population included all individuals aged 18 to 60 yrs. who participated in online study. All 308 individuals (229 women and 79 men) who completed the questionnaire online were selected.

The results were analyzed in two parts: descriptive and inferential. The parameters of percentage, frequency, mean and standard deviation were used in the descriptive section. In the inferential section, SPSS-23 statistical software was used to investigate the reliability of the instrument using internal consistency method, Cronbach's alpha and Guttmann’s lambda-2, and Factor-10 software and factor analysis using EFA were used to examine the construct validity. Confirmatory factor analysis (CFA) was performed using Lisrel-8.8 software.

### Corona Disease Anxiety Scale (CDAS)

To prepare Corona’s Anxiety Scale, various Items including AIDS Anxiety Questionnaire and Health Fear Questionnaire were studied and 23 items were selected. The questionnaire was given to 5 psychologists to examine the content validity of the Items. These subjects studied the Items in terms of concept and whether they cover all aspects of the subject as well as the form of the scale.

This scale has been designed and validated to measure the prevalence of the Coronavirus anxiety on Iran (Table 1). The final version of this instrument included 18 items and 2 factors, the items 1 to 9 measure the physical symptoms and items 10 to 18 measure the psychological symptoms. This instrument is scored on a 4-point Likert scale (never=0, sometimes=1, often=2 and always=3); therefore, minimum and maximum scores of the respondents in this scale are between 0 and 54. In this scale, high scores indicate a higher level of anxiety in individuals.

**Table 1:** The Corona Disease Anxiety Scale (CDAS)

***No***	***Question***	***Never***	***Sometimes Often***	***Always***
1	Thinking about Coronavirus makes me anxious			
2	I feel tense when I think about the Coronavirus threat.			
3	I am seriously worried about the prevalence of Coronavirus			
4	I am afraid of contracting Coronavirus			
5	I fear that I might contract Coronavirus anytime			
6	Minor symptoms make me think that I am contracting the virus, and I start checking myself			
7	I am concerned about transferring the virus to others around me.			
8	My anxiety about Coronavirus has interfered with my daily activities			
9	The mass medias focus on Coronavirus make me anxious			
10	Thinking about Coronavirus has interrupted my sleep			
11	I have lost my appetite because of thinking about Coronavirus			
12	I get a headache when I think about Coronavirus			
13	My body starts jittering when I think about Coronavirus			
14	I get goose bumps when I think about Coronavirus			
15	Coronavirus has become my nightmare			
16	I have less physical activity because of my fear of Coronavirus			
17	I find it hard to talk with others about Coronavirus			
18	I feel my heart beating when I think about Coronavirus			

### The General Health Questionnaire (GHQ)

The General Health Questionnaire (GHQ) is a screening device for identifying minor psychiatric disorders in the general population that within community or non-psychiatric clinical settings such as care or general medical out-patients. The 28-item General Health Questionnaire (GHQ–28) is a self-administered method to quantify the risk of developing psychiatric disorders. This instrument targets two areas – the inability to perform functions and the appearance of distress – to assess well-being in a person (7). The questionnaire evaluates somatic complain, severe depression, anxiety, insomnia and social dysfunction. This questionnaire was developed by Goldberg & Hillier and has been widely used in many countries in clinical and mental disorders. The reported Cronbach's alpha coefficient for the GHQ was a range of 0.82 to 0.86 in Iran (8). Maximum score is 84 in GHQ and is 21 in sub-scales and higher score indicates worsen wellbeing.

## Results

### Descriptive analysis

According to demographic findings, 80 individuals (26%) were female and 228 individuals (74%) were male, of whom 185 individuals (60.1%) were married and 123 (39.9%) were single. In terms of age, 59 individuals (2.19%) were in the age range of 18–25 yr, 89 (9.28%) were aged 26–33, 77 (25%) were aged 34–41; 48 (6.15%) were in the age range of 42–49 yr, 35 (4.11%) were aged 50–60. In terms of education, 57 individuals (18.4%) had high school or lower level, 105 individuals (34.1%) had bachelor degree, 90 individuals (29.3%) had master’s degree and 56 individuals (18.2%) had PhD. In the respondents' anxiety scores, independent t-test was used to examine the difference between gender and marital status. There was no significant difference in the anxiety variable with 99% confidence (*P*≥0.01) in terms of gender (*P*=0.669) and marital status (*P*=0.431). After the demographic findings were examined, the descriptive characteristics of "mean, standard deviation, skewness and kurtosis" of the questionnaire factors were reported in Table 2.

**Table 2:** Descriptive indicators of scale variables

*** Factor ***	*** Mean ***	*** SD ***	*** Skewness ***	*** Kurtosis ***
Psychological Symptoms	12.86	6.28	−0.13	−0.67
Physical Symptoms	4.88	5.64	1.37	1.30
The Total Questionnaire	17.74	11.05	0.60	−0.08

In order to examine the relationships between the components of the questionnaire, correlation matrix was used. Correlation matrix between the psychological symptoms and physical symptoms was 0.719, the Correlation between total scale and psychological symptoms was at 0.935 and the Correlation between the physical symptoms and total scales were at 0.919 (*P*≥0.01).

### Reliability

The reliability of this instrument was obtained by Cronbach's alpha for the physical symptoms (α =0.879), the psychological symptoms (α =0.861) and the total questionnaire (α =0.919). Moreover, the Guttmann’s λ -2 (1945) value were obtained for the physical symptoms (λ – 2 = 0.882), the psychological symptoms (λ - 2 = 0.864) and for the total questionnaire (λ - 2 = 0.922). (Table 3). To evaluate the reliability of the Items, the Loop method was utilized.

**Table 3:** Cronbach's alpha values of the whole questionnaire and its factors

*** Factor Name ***	*** Items of Each Factor ***	*** Number ***	*** Cronbach's Alpha ***	*** Guttman ***
Psychological Symptoms	1–9	9	0.879	0.882
Physical Symptoms	10–18	9	0.861	0.864
The Total Questionnaire		18	0.919	0.922

As observed from the results out of the Loop method in Table 4, all the items in the scale had proper discrimination (*rpbi*≥ 0.3), and by eliminating each item, the reliability of the questionnaire does not increase, hence all items in the scale remain in the analysis process. The internal consistency method, by Cronbach's alpha and Guttmann’s lambda 2 method was used.

**Table 4:** Reliability of the scale using the Loop method

*** Item ***	*** Mean ***	*** Discrimination ***	*** Standard Deviation ***	*** Cronbach's Alpha if Item Deleted ***
1	1.28	0.69	0.80	0.915
2	1.43	0.79	1.03	0.916
3	1.85	0.57	0.82	0.917
4	1.54	0.65	1.00	0.918
5	1.22	0.73	1.05	0.917
6	1.23	0.64	1.11	0.912
7	2.09	0.47	0.81	0.916
8	1.00	0.68	1.05	0.915
9	1.21	0.52	1.08	0.913
10	0.55	0.61	0.93	0.913
11	0.36	0.55	0.82	0.912
12	0.38	0.53	0.81	0.915
13	0.29	0.47	0.70	0.914
14	1.35	0.53	0.78	0.914
15	0.71	0.69	1.03	0.914
16	0.82	0.56	1.04	0.918
17	0.73	0.59	0.99	0.913
18	0.70	0.68	1.00	0.917

### Evaluating the Structural validity

To evaluate the structure validity, principal axis factoring and exploratory factor analysis (EFA) were utilized. The value of this statistic was KMO=0.937; hence, the assumption of the sample adequacy is confirmed. The value of the Bartlett sphericity statistic (2426.8) at the level of 0.01 with the degree of freedom of 153 was significant. After exploring the assumptions of exploratory factor analysis, to identify the number of extractable factors, a parallel analysis criterion, which is so accurate, was utilized.

As observed from the results of Table 5, two factors were proposed by parallel analysis method, conducted using the 2-factor command of analysis of scale items, and the extractable factors are presented in Table 6.

**Table 5:** Factors extracted of the questionnaire

*** NO ***	*** Real Eigenvalues ***	*** Mean of Random Eigenvalues ***	*** 95th Percentile Value ***
1	7.66 ^ ** ^	1.44	1.53
2	1.54 ^ ** ^	1.36	1.41
3	0.96	1.29	1.34
4	0.84	1.23	1.28
5	0.78	1.18	1.22
6	0.69	1.13	1.17
7	0.66	1.09	1.12
8	0.61	1.05	1.08
9	0.57	1.00	1.04
10	0.55	0.96	0.99
11	0.51	0.92	0.96
12	0.46	0.88	0.92
13	0.43	0.85	0.88
14	0.39	0.81	0.84
15	0.36	0.77	0.81
16	0.35	0.73	0.76
17	0.32	0.68	0.72
18	0.28	0.63	0.67

**Table 6:** Factor Analysis

*** Item ***		*** Psychological Symptoms ***	*** Item ***		*** Psychological Symptoms ***
1	0.703		1	0.688	
2	0.793	Composite	2	0.772	Composite
3	0.757	Reliability(CR)=83	3	0.738	Reliability(CR)=83
4	0.908		4	0.787	
5	0.835	Average Variance	5	0.803	Average Variance
6	0.706	Extracted =0.84	6	0.441	Extracted =0.84
7	0.648		7	0.311	
8	0.511		8	0.483	
9	0.382		9	0.526	

**Fig. 1:**
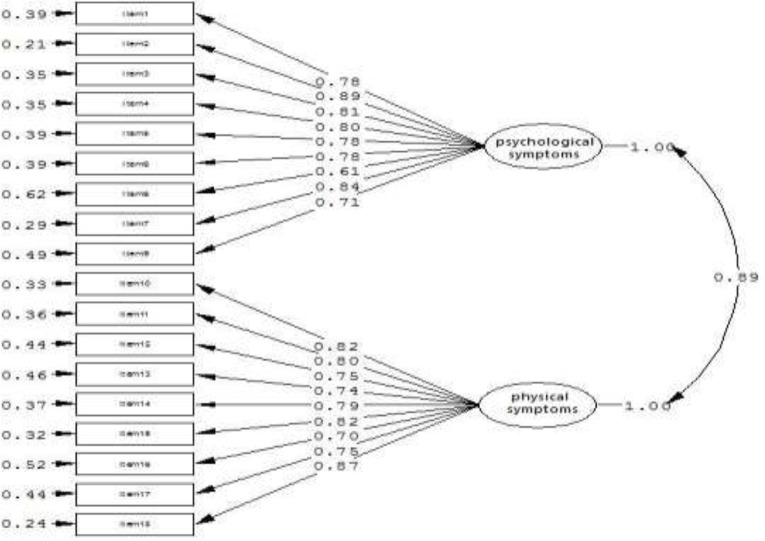
Two-factor scale model

As observed from the results of factor analysis in Table 6, psychological symptoms with 9 questions (1 to 9) and physical symptoms with 9 questions (10 to 18) were extracted. These two factors account for 51% of the total variance, of which 42% was related to psychological symptoms, and 9% was for physical ones, respectively. The value of composite reliability (CR) were obtained (cr= 0.83) for psychological symptoms, (cr= 0.84) for physical symptoms and (cr= 0.83) for the whole questionnaire. Besides, the average of the variance extracted (AVE) was obtained 0.61 for both factors and the total questionnaire. The conceptual model for the standardized coefficients is presented in Fig. 1.

As the outcomes of Table 7 relating to the correlation test between corona anxiety and general health components proved, there is a positive relationship between coronary anxiety and anxiety components (*r*=0.527), physical symptoms (*r*=0.418), social performance disorder (*r*=0.333), depression (*r*=0.269) and total score of lack of mental health (*r*=0.483), which is statistically significant with 99% confidence (*P* ≥0.01). As the coronary anxiety increases, the components of mental disorder will increase.

**Table 7:** The Correlation between CDAS and GHQ

*** Relationships Between Variables ***		*** Correlation ***	*** Significant ***
Corona Anxiety	Anxiety	0.527	0.001
	Physical symptoms	0.418	0.001
	Dysfunction in Social Functioning	0.333	0.001
	Depression	0.269	0.001
Total Mental Health Score		0.483	0.001

## Discussion

The purpose of this study was to developing and to construct and validate Corona Disease Anxiety Scale (CDAS) in order to provide its context for Iranian people. This study was conducted in two stages of construction and standardization.

The Corona Anxiety is a reliable and a valid scale for measuring and screening the anxiety in Iranian population. One of the most common indices of internal consistency is the Cronbach's alpha coefficient. In this study, the Cronbach's alpha was obtained from the Corona Anxiety Inventory was 0.91. Based on the criteria mentioned in the research, since alpha is above 0.7, it is very good. In the anxiety inventory known for other diseases, mainly Cronbach's alpha is less than 0.95. Before performing the analysis, the suitability of the data was assessed. The reliability of the Hamilton Anxiety Inventory was reported to be 0.81. Compared to that, the CDAS is more reliable. Exploratory factor analysis identified two factors (psychological and physical) for the questionnaire. The results of the correlations of the factors together with the whole scale showed that both factors had a positive and significant correlation with the total scale score.so this instrument is a reliable qestionnare.

Examination of the correlation coefficient showed coefficients of 0.4 and higher. The findings of the correlation test show that with increasing corona anxiety, non-mental health components increase. That is, as corona anxiety increases, physical symptoms, depression, anxiety, and social dysfunction increase. The questionnaire has good criterion validity. Anxiety has been widely acknowledged by researchers as an important and threatening variable of health (−11 9).

Previous similar research on the development of anxiety questionnaires in COPD patients has often emphasized the physical and psychological factors of these patients, which is consistent with the present study (10, 13–14).

Overall, the results of this study showed that Corona anxiety scale has good psychometric properties and can be used in Iran. The development of a standardized coronavirus anxiety questionnaire can provide new insights into the development and conduct of health psychology research and other areas of psychology and medicine. Since the main purpose of this questionnaire was to measure the level of anxiety of non-clinical people in relation to coronavirus, the results of this tool can be helpful for screening at the time of outbreak and even after the disease has subsided.

## Conclusion

Because the subject is new, these data need to be replicated with larger samples in future research. It is also suggested to use other validity assessment methods for this scale. Because of the widespread prevalence of the disease in Iran at the time of the study, standard and in-person sampling was not possible. After reducing the prevalence of the disease, it is recommended to standardize this scale in different classes of society.

## Ethical considerations

Ethical issues (Including plagiarism, informed consent, misconduct, data fabrication and/or falsification, double publication and/or submission, redundancy, etc.) have been completely observed by the authors.
